# Genome Sequence of *Arenibacter algicola* Strain TG409, a Hydrocarbon-Degrading Bacterium Associated with Marine Eukaryotic Phytoplankton

**DOI:** 10.1128/genomeA.00765-16

**Published:** 2016-08-04

**Authors:** Tony Gutierrez, William B. Whitman, Marcel Huntemann, Alex Copeland, Amy Chen, Nikos Kyrpides, Victor Markowitz, Manoj Pillay, Natalia Ivanova, Natalia Mikhailova, Galina Ovchinnikova, Evan Andersen, Amrita Pati, Dimitrios Stamatis, T. B. K. Reddy, Chew Yee Ngan, Mansi Chovatia, Chris Daum, Nicole Shapiro, Michael N. Cantor, Tanja Woyke

**Affiliations:** a School of Life Sciences, Heriot-Watt University, Edinburgh, United Kingdom; b Department of Microbiology, University of Georgia, Athens, Georgia, USA; c DOE Joint Genome Institute, Walnut Creek, California, USA

## Abstract

*Arenibacter algicola* strain TG409 was isolated from *Skeletonema costatum* and exhibits the ability to utilize polycyclic aromatic hydrocarbons as sole sources of carbon and energy. Here, we present the genome sequence of this strain, which is 5,550,230 bp with 4,722 genes and an average G+C content of 39.7%.

## GENOME ANNOUNCEMENT

*Arenibacter algicola* strain TG409 was isolated from a laboratory culture of the marine diatom *Skeletonema costatum* (CCAP 1077/1C) by enrichment with polycyclic aromatic hydrocarbons (PAHs) as the sole carbon source (1). The strain represents the first *Arenibacter* species associated with a laboratory culture of *S. costatum* and shows the ability to degrade PAHs as a sole source of carbon and energy (1). Notably, work with this and other *Arenibacter* type strains has shown that PAH degradation may be a shared phenotype among members of this genus (1). Strain TG409 is a strictly aerobic and nonmotile rod-shaped bacterium that is associated with the cosmopolitan marine diatom *S. costatum*. It produces amphiphilic biopolymers (e.g., biosurfactants) on the cell surface that may facilitate attachment to oil droplets and increase the bioavailability of hydrocarbons (1).

Here, we report the genome sequence of *Arenibacter algicola* strain TG409. Genomic DNA was isolated, and the sequence generated at the Department of Energy (DOE) Joint Genome Institute (JGI) using the Pacific Biosciences (PacBio) technology. A Pacbio SMRTbell library was constructed and sequenced on the PacBio RS platform, which generated 292,099 filtered subreads totaling 1074.1 Mbp. All general aspects of library construction and sequencing performed at the JGI can be found at http://www.jgi.doe.gov. The raw reads were assembled using HGAP (version: 2.1.1) (2). The final draft assembly produced three scaffolds containing three contigs totaling 5.6 Mbp in size and input read coverage of 185.2×.

Project information is available in the Genomes Online Database (3). Genes were identified using Prodigal (4), followed by a round of manual curation using GenePRIMP (5) as part of the JGI’s microbial annotation pipeline (6). The predicted coding sequences (CDSs) were translated and used to search the National Center for Biotechnology Information (NCBI) nonredundant database and the UniProt, TIGRFam, Pfam, KEGG, COG, and InterPro databases. The tRNAScanSE tool (7) was used to find tRNA genes, whereas rRNA genes were found by searches against models of the rRNA genes built from SILVA (8). Other noncoding RNAs, such as the RNA components of the protein secretion complex and the RNase P, were identified by searching the genome for the corresponding Rfam profiles using INFERNAL (http://infernal.janelia.org). Additional gene prediction analysis and manual functional annotation was performed within the Integrated Microbial Genomes–Expert Review (IMG ER) platform (https://img.jgi.doe.gov/) developed by the Joint Genome Institute, Walnut Creek, CA, USA (9).

The complete genome sequence length was 5,550,230 bp with a G+C content of 39.7%. The genome contains 4,722 genes (4,649 protein-coding genes) with functional predictions for 3,538 of them. A total of 73 RNA genes were detected. Other genes, characteristic for the genus, are given in the IMG database (9).

### Nucleotide sequence accession number.

The draft genome sequence of *A. algicola* strain TG409 obtained in this study was deposited in GenBank as part of BioProject no. PRJNA224116, with individual genome sequences submitted as whole-genome shotgun projects under the accession no. JPOO00000000.
